# Risk of inadequate protein and micronutrient intakes in patients with PKU with an increased phe-tolerance: Impact of a micronutrient-dense protein substitute

**DOI:** 10.1016/j.ymgmr.2025.101264

**Published:** 2025-10-04

**Authors:** Carmen Rohde, Denise Leonne Hofman, Ira Klawon, Frank Rutsch, Iris Rodenburg, Francjan van Spronsen, Alena Gerlinde Thiele, Willie Woestenenk, Skadi Beblo

**Affiliations:** a Clinic for Pediatrics and Adolescent Medicine, University Hospital Leipzig, Leipzig, Germany; b Danone Research & Innovation, Utrecht, the Netherlands; c Clinic for Pediatrics and Adolescent Medicine, University Hospital Münster, Münster, Germany; d Division of Metabolic Diseases, Beatrix Children's Hospital, University Medical Centre Groningen, Groningen, the Netherlands

**Keywords:** Phenylketonuria, Relaxed diet, Protein, Amino acid, Micronutrient, DHA

## Abstract

**Background:**

Patients with phenylketonuria (PKU) with higher phenylalanine (phe)-tolerance may have inadequate diets due to high-quality protein restriction and reduced intakes of nutrient-fortified phe-free, tyrosine (tyr)-enriched protein substitutes (PS).

**Methods:**

Open-label, 24-week, multi-centre intervention assessed the impact of a once-daily, micronutrient-dense PS on nutrient intakes in patients with an increased phe-tolerance. Subjects >12 years, consuming 0–25 g protein/day from a regular PS, were recruited. Diet records and fasting blood samples were collected at baseline and study end.

**Results:**

11/13 subjects, 15-49y (5 males), with 6/11 taking tetrahydrobiopterin dihydrochloride (BH4) and/or a regular PS, completed the study. At baseline, protein and some essential amino acids (EAA) intakes were below recommendations in 4/11 and 3/11 subjects, respectively. Vitamins A, B2, B12, D, calcium, iron, iodine, zinc and magnesium intakes were below recommendations for 7/11. After the intervention, 2/11 had total protein intakes below recommendations, while 11/11 met EAA recommendations. Micronutrient intakes improved to ≥90 % of recommendations for all subjects except for vitamin B12 (4/11) and phosphorus (1/6). Docosahexaenoic acid (DHA) intakes increased. Blood phe levels remained stable.

**Conclusion:**

Patients with PKU who have relaxed their phe-restricted diet are at risk of insufficient nutrient supply, and dietary counselling should be offered. Consuming the study PS increased nutrient intakes closer to dietary recommendations.

## Highlights

• Patients with PKU on a relaxed phe-restricted diet are at risk of insufficient nutrient supply
• The study protein substitute increased nutrient intakes closer to dietary needs.
• Patients blood Phe levels remained stable, and DHA intake improved.

## Graphical abstract

**Figure:**
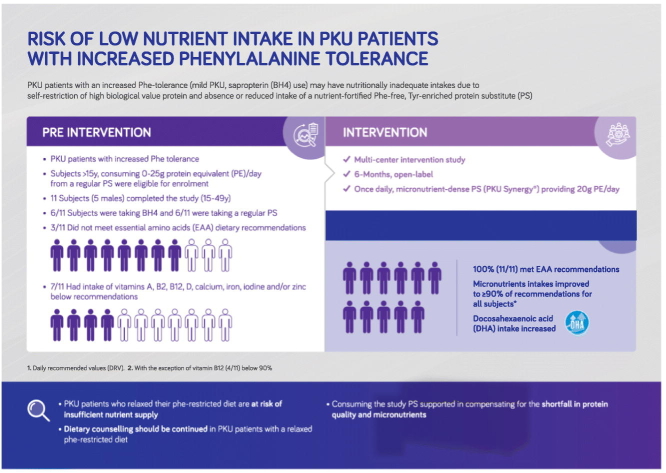

## 1 Introduction

Phenylketonuria (PKU) is a rare inborn error of metabolism (approximately 1 in 24,000 newborns globally) caused by an insufficiency or absence of the enzyme phenylalanine hydroxylase (PAH), which is required to convert phenylalanine (phe) to tyrosine (tyr) [1]. If untreated, the defect in PAH activity leads to the accumulation of toxic levels of phe especially in the blood and brain, resulting in irreversible intellectual disability, motor deficits, and abnormal developmental behavioral and psychiatric outcomes. With dietary management, the symptoms of untreated PKU can be largely avoided, although minor cognitive deficits may be apparent [2]. Dietary management requires a strict, low natural-protein diet which is typically supplemented with a phe-free protein substitute (PS) and low protein food to meet nutritional requirements for all non-phe amino acids and micronutrients. Due to advances in treatment (e.g., cofactor treatment with tetrahydrobiopterin dihydrochloride (BH4)), or higher phe tolerance, some patients with PKU can relax their diet by increasing intakes of natural protein and ceasing/reducing intakes of their PS [3]. Dietary relaxation means patients can include previously excluded foods, such as regular bread and pasta. However, in the absence of specific guidelines for managing relaxed diets, clinical practise varies across centres.

In adolescence and adulthood, changing established dietary habits to incorporate unfamiliar food tastes and textures can be difficult for patients [[4], [5], [6]]. Studies on the dietary habits of patients with PKU following dietary relaxation have observed differences in eating patterns between this group and the general population [4,5]. Thiele et al. [4] found that the intake of animal-origin foods, in particular fish and dairy products, and vegetables and fruit were markedly lower than healthy controls, and that bread, pasta and cereal products were consumed in greater quantities. Consequently, those with PKU on relaxed diets may be at risk of poorer intakes of some nutrients, which may in turn affect their nutritional status. In a systematic review, Ilgaz et al. [3] observed that poor micronutrient intakes were associated with diets that had not been completely relaxed, and PS intakes had been reduced by at least half of the patient's usual prescription. Intakes of the long chain polyunsaturated fatty acid (LC-PUFA), docosahexaenoic acid (DHA), may also be inadequate due to poor intakes of sources such as fatty fish [4], although one study found that DHA status was adequate in patients with PKU on a relaxed diet [7].

The micronutrient requirements of patients with PKU are the same as those of healthy individuals [8,9]. Because nutrient intake from food sources in a relaxed PKU diet may be insufficient to meet intake recommendations, patients may continue to require a PS or micronutrient supplement [[3], [4], [5]]. Regular PS will provide a much lower contribution to overall micronutrient intakes when consumed in the smaller volumes typical of patients on a relaxed diet, potentially increasing the risk of deficiencies in key nutrients such as calcium of iron. For example, per 20 g protein equivalent, regular PS typically provides 398–560 mg calcium, and 6.6–7.2 mg iron, whereas the micronutrient-dense PS specifically designed for relaxed-diet PKU patients delivers 840 mg calcium, and 10 mg iron [10] (see Table A1 for nutritional composition of the study product, as well as the compositions of regular PS). Green et al. [11] found that a once/day micronutrient-dense PS tailored to the needs of PKU patients on a relaxed diet provided micronutrient intakes that met recommendations over 4 weeks. The aim of the study reported here was to assess the impact of the same once/day micronutrient-dense PS (PKU Synergy® by Nutricia, Danone) over 24 weeks on intakes of selected nutrients and nutrient status of adolescents (≥ 12 years) and adults with PKU with an increased Phe-tolerance/intake.

## 2 Materials and methods

Potentially eligible subjects from 3 metabolic centres in Europe (Leipzig, Germany; Münster, Germany; Groningen, The Netherlands) were invited to participate in a 24-week, open-label intervention study investigating the impact on nutrient intakes of a once-a-day powdered PS with a tailored amino acid and micronutrient profile. All required research ethics committee and site-specific approvals were obtained (local site-specific EC numbers: 255/19-IK [Leipzig], 2019–014-f-S [Münster], 2019/283 [Groningen]; Trial registered at ClinicalTrials.gov ID: NCT03777826). Written, informed consent from subjects (and parents, where appropriate) was sought and obtained. The study was undertaken in line with ICH-GCP and the principals outlined in the Declaration of Helsinki.

Subjects eligible for the study were over 12 years of age (non-pregnant, non-lactating, not intending to become pregnant during the study), diagnosed with PKU at newborn screening and treated with a phe restricted diet before 3 months of age, had an increased tolerance for phe (due to a mild phenotype and/or managed with/without BH4 treatment) and therefore a more relaxed PKU diet (i.e., either no longer taking an PS or taking no more than 25 g protein from a PS/d). Subjects managed with BH4 treatment were required to have remained on a stable regimen for at least 26 consecutive weeks prior to visit 1. If subjects were taking a regular PS, they were required to consume it daily for at least 26 consecutive weeks prior to visit 1. Excluded subjects were those who had taken the test product within 6 weeks of study entry and those with concurrent conditions or medications that could interefere with outcome parameters or safety. Subjects replaced their current PS (if taken) with a single serving/d of the study product (PKU Synergy; 20 g protein/d) for the 24-week intervention period. Other than incorporating the study product into their diet, subjects were instructed to maintain their usual dietary regime.

Subjects kept diet records of food, beverages and PS consumed (in household measures) over 3 consecutive days (2 mid-weekdays, 1 weekend day) at baseline (day 1–3) and during the final study week. These records were used to assess intakes of energy, protein, essential amino acids (EAAs), DHA, and micronutrients (vitamins A, B1, B2, B12, C and D, calcium, phosphorus, iron, magnesium, iodine, selenium, zinc). Diet records were analysed using commercial dietary analysis software that draws on food composition data in Bundeslebensmittelschluessel, version 3.02 (BLS 3.02) (Leipzig and Münster, Germany) and Dutch Food Composition Database (NEVO) (RIVM Bilthoven, The Netherlands). Intakes of protein and micronutrients were compared with European recommendations for the general population [12,13] and intakes of EAAs with WHO recommendations [14]. Study product intake was recorded to assess compliance, and acceptability evaluated by questionnaire at weeks 1, 12 and 24. Adverse events were monitored throughout the study and tolerance by questionnaire at baseline, weeks 1, 12 and 24. Subjects completed a detailed questionnaire on the acceptability of the product.

Fasting venous blood samples were collected at baseline and study end for the assessment of EAAs and markers of vitamins B12 and D, folic acid, selenium, iodine, zinc and iron. Blood samples were centrifuged, aliquoted and stored at a − 80 °C at the site before analysis. Additionally, bi-weekly dried blood spots taken at home were collected for the assessment of phe levels. Blood analyte results were compared to validated reference intervals used by the respective laboratories.

### 2.1 Data handling and statistics

Data were collected in the electronic data capture system, Viedoc 4. The questionnaire data were collected in ViedocMe. Electronic captured data were validated using automated edit checks and data validation steps. Because of the small number of subjects recruited to the study, descriptive statistics only are reported. For the same reason, robustness and sensitivity analyses were not conducted. As the data was not normally distributed, medians and IQRs are presented for the group data.

## 3 Results

Thirteen subjects (6 males) aged 15–49 years were recruited. Eleven subjects (5 males with a phe tolerance of >1300 mg/d) completed the study. Two subjects who experienced moderate nausea withdrew after 5 days (at baseline, one subject had been taking a regular PS prior study entry, and the other subject had not). The baseline characteristics of the population who completed the study are included in Table 1.

**Table 1:** Baseline characteristics of study sample (*n* = 11).

Gender	6 females, 5 males
Age in years, median (IQR)	19 (15–35)
Annual phe level in year pre-enrolment in μmol/L, median (IQR)	494 (372–720)
Phe tolerance in mg/d, range	1300–3000
Use of PS + BH4 at baseline (n)	2
Use of PS only at baseline (n)	4
Use of BH4 only at baseline (n)	4
Use of neither PS nor BH4	1
Where taken, protein in g from PS at baseline, median (IQR)	20.5 (10.0–21.0)
Where taken, BH4 dose in mg/kg bodyweight/d, median (IQR)	12.9 (8.8–14.0)
Body mass index (BMI) (kg/m^2^), median (IQR)	23.5 (19.1–24.3)
Underweight (n, %)	1 (9 %)
Normal weight (n, %)	7 (64 %)
Overweight (n, %)	3 (27 %)

Notes: * BMI ranges for adults: normal/healthy between 18.5 and 24.9; overweight between 25 and 29.9; obese over 30. BMI range for children <18 years of age: age-specific score determined using the BMI centile charts produced by the WHO.

### 3.1 Protein and essential amino acids intakes

Average protein intakes from food sources alone were similar at baseline and study end, although there was a wide range of protein intakes at both study timepoints (protein intakes from all sources ranged from 35 to 86 g/d at baseline and 51–109 g/d at study end) (Table 2).

**Table 2:** Median (IQR) intakes of protein and phe intakes at baseline and study end by compliant subjects (*n* = 11).

Nutrient	Baseline		End of intervention	
	From food sources only	From all sources	From food sources only	From all sources
Protein, g/d	52.2 (45.3–60.6)	66.1 (56.4–72.5)	48.2 (43.1–65.2)	68.0 (63.1–85.2)
Protein, g/kg bw/d	0.8 (0.7–1.0)	1.0 (0.7–1.2)	0.8 (0.6–1.1)	1.1 (0.9–1.5)
Phe intake, mg/d	2413 (1988–2847)	2413 (1995–2850)	1942 (1428–2984)	1945 (1431–2987)
Phe intake, mg/kg bw/d	34 (28–50)	34 (28–50)	32 (18–47)	32 (18–47)

**Fig. 1:**
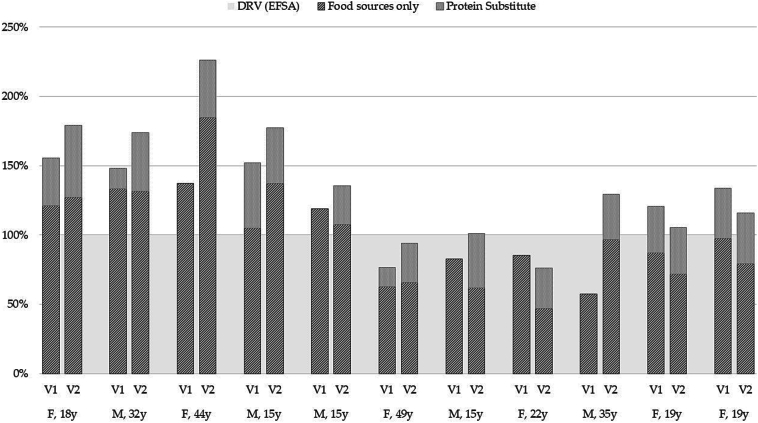
Protein intake at baseline (V1) and study end (V2) for individual subjects, as a percentage of EFSA Dietary Reference Values.

At baseline, intakes of protein from food sources alone met European food safety authority (EFSA) recommendations for 5/11 subjects (Fig. 1). Protein intakes from all sources (food and, if taken, PS) met EFSA recommendations for all but 4 subjects (58 % - 85 % of EFSA recommendations), one of whom was taking a PS that provided 10 g protein/d.

During the intervention, all subjects took 1 serving (20 g protein) of the study product per day. At study end, protein intakes from all sources met EFSA recommendations for all but 2 subjects (2 females, 74 % and 96 % of recommendations; Fig. 1).

**Fig. 2:**
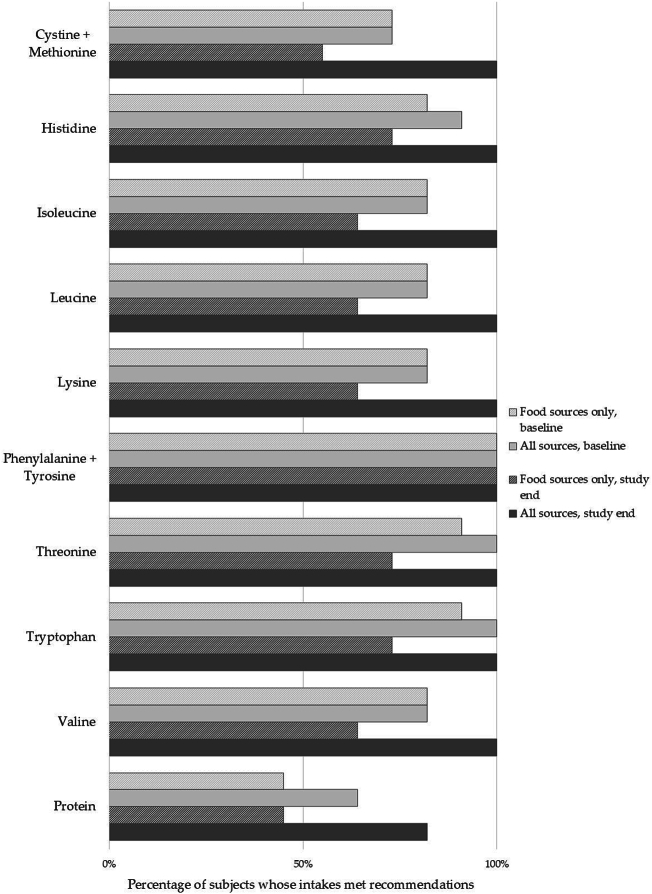
Percentage of subjects whose intakes of protein and essential amino acids met or. exceeded WHO intake recommendations.

At baseline, 3 of the 4 subjects who had protein intakes from all sources below EFSA recommendations (Fig. 1) also had EAA intakes below WHO recommendations (*n* = 3 cystine + methionine, *n* = 2 for isoleucine, leucine, lysine, *n* = 1 for histidine). Intake assessments at study end showed that the study product and foods combined provided intakes that met all EAA recommendations for all subjects (Fig. 2).

### 3.2 Vitamin, mineral, trace element and DHA intakes and biochemical levels

**Fig. 3:**
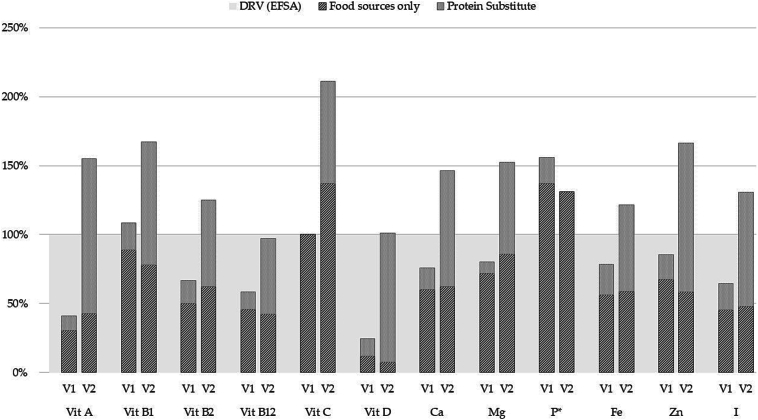
Median micronutrient intakes at baseline (V1) and study end (V2), as a percentage of EFSA Dietary Reference Values. No information on phosphorus intakes from dietary sources for 5 subjects. Data presented for 6 subjects.

The data presented in Table 3 show that, at baseline, most subjects had micronutrient intakes from all sources that were below population recommendations. Intakes of vitamins A, B2, B12, D, calcium, magnesium, iron, zinc and iodine were below recommendations for between 7 and 11 subjects at baseline. Only for vitamin B1 and phosphorus were intake recommendations met by most subjects at baseline (Table 3). At study end, intake recommendations were met by all subjects for vitamins A, B1, C, calcium, magnesium, iodine, and those few subjects whose intakes were below the DRVs for vitamins B2, B12, D and phosphorus, iron and zinc were only marginally below (Table 3). Median micronutrient intakes for the group, expressed as a percentage of EFSA recommendations, are presented in Fig. 3 (see Supplementary Table S1: daily intake of protein, essential amino acids and micronutrients of individual subjects). For selenium, information on the contribution from food sources was available for only 2 subjects and therefore data for this nutrient has been excluded. The median intake of DHA increased from 20 mg/d at entry to 113 mg/d at study end (EFSA recommendation for DHA + eicosapentaenoic acid (EPA) = 250 mg/day).

**Table 3:** Intakes of micronutrients and DHA from all sources at baseline and study end by compliant subjects (*n* = 11).

Nutrient	Daily intakes, median (IQR)		Intakes below EFSA DRVs, number of subjects (range of intakes expressed as a %DRV)	
	Baseline	Study end	Baseline	Study end
Vitamin A, μg	534 (295–576)	1029 (910–1141)	10 (8–95 %)	0
Vitamin B1, mg	1.2 (1.1–1.3)	1.5 (1.5–1.7)	3 (75–97 %)	0
Vitamin B2, mg	1.3 (0.9–1.4)	2.0 (1.7–2.2)	11 (11–98 %)	2 (94–95 %)
Vitamin B12, μg	2.8 (1.9–3.5)	3.9 (3.3–6.8)	9 (39–90 %)	6 (63–97 %)
Vitamin C, mg	131 (69–187)	200 (172–228)	4 (6–88 %)	0
Vitamin D, μg	4.8 (1.9–8.6)	15.1 (14.5–16.9)	11 (4–65 %)	5 (94–99 %)
Calcium, mg	850 (656–904)	1522 (1381–1594)	10 (29–95 %)	0
Magnesium, mg	280 (226–353)	457 (384–502)	7 (59–98 %)	0
Phosphorus, mg*	743 (557–1058)	720 (630–844)	1 (87 %)	1 (70 %)
Iron, mg	13.8 (8.8–15.0)	17.8 (16.5–21.7)	9 (50–97 %)	1 (98 %)
Zinc, mg	10.0 (7.4–11.9)	16.1 (15.3–17.6)	8 (35–96 %)	1 (95 %)
Iodine, μg	122 (67–143)	196 (165–205)	7 (22–88 %)	0
DHA, mg	20 (20–165)	113 (110–175)	-^†^	-^†^


Notes: * No information on phosphorus intakes from dietary sources for 5 subjects. Data presented for 6 subjects.

^†^ Recommendation is for DHA + EPA (250 mg/d), not DHA alone.

Blood analyte results were available for 9/11 subjects at baseline and at study end for all subjects (n = 11). Blood levels of markers of zinc, iron, folate, calcium and iodine were within respective laboratory reference intervals at baseline and study end. One subject for each of plasma vitamin B12, vitamin D and selenium were below the reference interval at baseline, with levels improving by study end (Table 4). Plasma DHA (% of total fatty acids) was measured for 6 subjects from two of the three study sites. Levels increased from a range of 1.0–1.5 % at baseline to 1.3–2.1 % at study end.

**Table 4:** Blood results range for plasma nutrient levels at baseline and end of study compared to laboratory reference intervals.

Analyte	Reference Interval at local laboratories *	Baseline, range (number subjects)	Study end, range (number subjects)
Plasma vitamin B12, pmol/L	130–700 (6 subjects)	112–343 (*n* = 4)^1,2^	171–384 (*n* = 6)
	145–569 (5 subjects)	205–507 (*n* = 5)	244–419 (n = 5)
Plasma 25(OH)D, nmol/L	>50 (6 subjects)	16–77 (n = 4)^1,3^	46–82 (n = 6)^4^
	>20 (5 subjects)	48.5–61.5 (n = 5)	47.25–96.0 (n = 5)
Plasma selenium, μmol/L	0.8–1.8 (6 subjects)	0.72–0.94 (n = 4)^1,5^	0.8–1.0 (n = 6)
	0.46–1.36 (5 subjects)	0.56–0.81 (n = 4)^6^	0.62–1.13 (n = 5)
Plasma DHA % (mg/L)	No reference interval available	1.0–1.5 (22.3–38.7) (n = 4)^7^	1.3–2.1 (26.9–57.1) (n = 6)^8^

Notes: * reference interval from validated laboratories.

Levels below reference intervals: ^1^*n* = 2 no baseline data available, ^2.^*n* = 1 at 112 pmol/L, ^3.^ n = 1 at 16 nmol/L, ^4.^ n = 1 at 46 nmol/L, ^5.^ n = 1 at 0.72 μmol/L, ^6.^ n = 1 no baseline data available, ^7.^*n* = 7 no baseline data available, ^8.^ n = 5 no end visit data available.

### 3.3 Phe control and plasma amino acids (AAs)

**Fig. 4:**
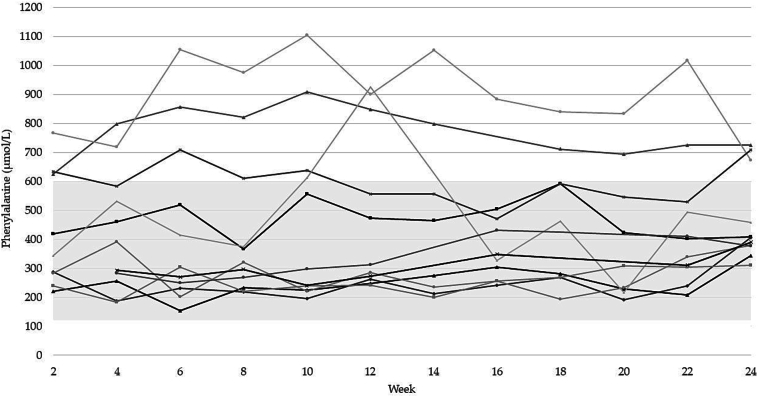
Biweekly plasma phe levels for each subject between baseline and study end.

Phe control remained stable over the 24-week period, except for one patient whose Phe levels spiked around week 12, likely due to diarrhea. Five subjects remaining below 600 μmol/L throughout the study (Fig. 4). Plasma EAAs levels were within normal ranges for all subjects, except for low tyrosine and leucine for two patients despite adequate protein intakes.

### 3.4 Acceptability of the study product

Compliance with the study product was reported to be between 90 and 100 % based on number of sachets taken per week for the 24-week intervention. The average overall acceptability rating was 4.9 (range 1–8) on a scale of 1–10 (1: ‘extremely dislike’ and 10: ‘extremely like’). Adverse events reported during the study were predominantly gastrointestinal (11 out of 12 events) i.e., flatulence, abdominal distension, diarrhea or nausea. No changes to PS intake were required or requested because of these adverse events. Overall, the product was safe with no serious adverse events reported.

## 4 Discussion

This study investigated the nutritional quality of the diets of patients with PKU who had a higher phe tolerance and the effect of a once-a-day, micronutrient-dense PS on protein and nutrient intakes. It was observed that the extent of relaxation of natural protein intakes varied considerably, reflecting the real-world practises of PKU patients on a relaxed diet across different centres, with just under half of the study subjects (5/11) having protein intakes from foods sources alone that met EFSA intake recommendations [13] at both baseline and study end. Including the contribution from a regular PS, where taken, EFSA protein recommendations were achieved by 7/11 subjects at baseline. The quality of protein was sub-optimal at baseline for some, with 3/11 subjects having intakes of 2 or more of the EAAs below WHO intake recommendations [14], despite one of these subjects taking a regular PS. At study end, including the contribution from the study product, protein intakes from all sources for 9/11 subjects were at or above EFSA recommendations [13]. Furthermore, EAA intakes from all sources met population intake recommendations [14] for all subjects at the end of the intervention. Plasma EAA levels were within reference intervals for all subjects at baseline and study end, with only two exceptions, which may have been due to extended fasting times before the sample was taken [15]. The once-daily intake of the study product simplifies dietary management and enhances adherence, particularly in adults with PKU. Although free amino acids may cause transient plasma Phe fluctuations, these are likely buffered by natural protein intake throughout the day [16]. This was demonstrated in the current study, where Phe concentrations were maintained within a stable range throughout the study period.Changes in dietary patterns and food types in a relaxed PKU diet can affect its nutritional quality [4,5,17], with several studies observing lower protein and nutrient intakes compared with recommendations, especially if the PS was discontinued or intakes were substantially reduced [5,6,17,18]. Rohde et al. [5] reported that patients with PKU taking a relaxed diet are at risk of an insufficient nutrient supply if they have: no substitution with PS; a protein supply less than 0.5 g/kg from PS; or total protein supply <120 % of recommendations. PKU-specific recommendations for protein intakes are usually higher (around 120–140 %) than population recommendations to allow for the low biological value of the allowed natural protein sources in the diet and the synthetic nature of protein in the PS [9,19]. Vegetable and cereal-based protein sources typically have a low biological value compared to animal-origin protein sources [14]. The higher intake of these low biological value protein sources, coupled with a lower protein intake in a relaxed PKU diet [6,20], may put patients at risk of poor EAA intakes.

Other studies have observed that patients with PKU on a relaxed diet did not meet intake recommendations for a range of micronutrients (vitamin D, B-vitamins, folic acid, iron, calcium, iodine, selenium, zinc) [4,5,17,18,21]. Protein-rich foods are good sources of a wide range of nutrients, however patients who have relaxed their PKU diet often self-restrict such foods. It has been recommended that patients on a relaxed diet may benefit from continued use of a fortified PS or a separate multivitamin supplement [4,5,18]. As observed in this study, despite almost half of the group taking reduced amounts of a regular micronutrient-fortified PS at baseline, most subjects did not meet intake recommendations for vitamins A, B1, B2, B12, C, D, calcium, magnesium, iron, zinc and iodine. Regular PS for a phe-restricted diet are designed to provide nutritionally appropriate intakes of micronutrients when these provide around 70–85 % of total protein intake. In this study, reduced intakes of the regular PS taken with a relaxed diet resulted in micronutrient intakes that were below recommendations for most subjects.

The PS used in this study (PKU Synergy® Nutricia, Danone) was developed to provide intakes of vitamins, minerals and DHA to meet the expected shortfall in these nutrients, but in a lower volume than traditional PS. Variability in nutrient intake increases may reflect differences in nutrient levels between the study product and standard protein substitutes. In this study, intakes of vitamins A, B1, C, calcium, magnesium, iron, zinc and iodine substantially improved, with all subjects meeting intake recommendations [13] at study end. Intakes of vitamins B2, B12 and D, iron and zinc improved for all subjects, although intakes were not above intake recommendations for all subjects at study end. These findings concur with an earlier intervention using the same study product by Green et al. [11], who observed that micronutrient intakes improved with PKU Synergy® in patients with PKU following an unrestricted diet. These improvements in micronutrient intake are particularly relevant given the differences in bioavailability between synthetic and food-bound nutrient sources, as it is acknowledged that micronutrients in amino acid-based formulas may exhibit different absorption profiles compared to food-bound forms [22]. However, the intake of natural protein throughout the day may help balance these differences.

Despite nutrient intakes being below recommendations for several nutrients at baseline, blood nutrient analytes were found to be within laboratory reference intervals for zinc, iron, folate, calcium and iodine at both baseline and study end. However, blood analytes for vitamin B12, vitamin D and selenium were towards the lower limit of the range which may indicate a functional deficiency or sub-optimal status [23,24] for some subjects. Vitamin B12 is important in central nervous system function, and levels of vitamin B12 for 4 subjects at baseline were below recognized thresholds for sub-clinical deficiency (frank’ deficiency <148 pmol/L; mild deficiency (148-260 pmol/L). Adequate levels of vitamin D are required for bone health and immune function, and two subjects had levels below deficiency thresholds [24] at baseline. At baseline, compared with recommended functional ranges [24], 3 subjects had selenium levels below those considered adequate (60 μg/L). Levels of vitamin B12, D and selenium improved by study end, but were below these (sub-clinical) deficiency thresholds for some subjects. Other studies [15,16,19,25,26] that assessed blood analytes among patients who had relaxed their phe-restricted diet did not report widespread evidence of poor nutrient status.

In addition to micronutrients, DHA intakes improved substantially, particularly for subjects with poor intakes (lowest baseline intake increased from 20 mg to 110 mg/day while taking the study PS). Given the poor fish intake reported for patients on a relaxed diet [4] and the importance of DHA in neurological function, this increase in DHA intake makes a significant contribution towards meeting the EFSA recommendation [13] for DHA + EPA (250 mg/day or 1 to 2 fatty fish meals per week). This increase in DHA intake was reflected in an increase in plasma DHA levels.

Structured nutritional counselling during the introduction of protein-containing foods and the management of dietary imbalances in patients with PKU with an increased protein tolerance is critical for guiding balanced food choices [27]. However, it may not fully overcome entrenched dietary habits, particularly in individuals with long-standing restrictive eating patterns. In this study, patients taking a PS that is tailored to a relaxed diet were compliant over 24 weeks with the once/day supplement. Protein and EAA intakes increased, and the PS successfully ‘closed the gap’ in nutrient intakes from dietary sources. Overall, the product was safe and generally well tolerated. Overall acceptability ratings were variable, which may reflect individual taste preferences for an AA-based PS.

### 4.1 Strengths and limitations

This was the first multi-centre, multi-country, long-term intervention in a group of PKU patients on a relaxed diet. Despite the large size of the participating centres, recruitment was limited due to the niche nature of PKU patients on a relaxed diet. The size of the study sample (13 recruited, 11 completed) is a limitation of our study and affects the generalizability of the results to the wider population of patients with PKU on a relaxed diet. Nevertheless, our findings concur with previously published research on nutrient intakes and nutritional status of this sub-group of the PKU population.

## 5 Conclusions

Patients with PKU who have relaxed their Phe-restricted diet are at risk of insufficient supply of protein, EAAs, micronutrients and DHA. Although the subjects in this study were taking a more relaxed natural protein diet, their protein requirements may be higher than the general population to meet their EAA requirements given their dependence on low biological value protein sources. Baseline results from this study suggest that intakes from a regular PS of ≤25 g protein/day provide inadequate intakes of protein, EAAs, micronutrients and DHA.

This patient population should receive dietary counselling to ensure the nutritional adequacy of the diet. The micronutrient-dense PS in this study provided protein and micronutrient intakes which, combined with the rest of the diet, were sufficient to meet intake recommendations and, where low, promoted improved blood micronutrient status.

The following is the supplementary data related to this article.

Supplementary Table S1 Daily intake of protein, essential amino acids and micronutrients of individual subject. Supplementary Table S1

## CRediT authorship contribution statement

**Carmen Rohde:** Writing – review & editing, Writing – original draft, Methodology, Investigation, Conceptualization. **Denise Leonne Hofman:** Writing – review & editing, Writing – original draft, Project administration, Conceptualization. **Ira Klawon:** Writing – review & editing, Investigation, Conceptualization. **Frank Rutsch:** Writing – review & editing, Investigation, Conceptualization. **Iris Rodenburg:** Writing – review & editing, Investigation, Conceptualization. **Francjan van Spronsen:** Writing – review & editing, Writing – original draft, Methodology, Investigation, Conceptualization. **Alena Gerlinde Thiele:** Writing – review & editing, Investigation, Conceptualization. **Willie Woestenenk:** Writing – review & editing, Writing – original draft, Project administration, Conceptualization. **Skadi Beblo:** Writing – review & editing, Writing – original draft, Methodology, Investigation, Conceptualization.

## Informed consent statement

Informed consent was obtained from all subjects involved in the study.

## Institutional review board statement

The study was conducted in accordance with the Declaration of Helsinki, and site-specific approvals were obtained from local research ethics committees at the 3 participating sites.

## Funding

This research was supported by 10.13039/100007773Danone Research & Innovation.

## Declaration of competing interest

C.R. and S.B. have received research grants from Danone / Nutricia. F.S. has received research grants, advisory board fees, and/or speaker's honoraria from Danone / Nutricia. D.H. and W.W. are employees of Danone Research & Innovation. The other authors have no conflicts of interest to declare. The funder was involved in the design of the study, the collection, analyses or interpretation of data, the writing of the manuscript and the decision to publish the results.

## Figures and Tables

**Table A1:** Nutritional composition of the investigational product (PKU Synergy®) and the compositions of regular protein substitutes [10].

	PKU Synergy® Per single sachet of powder (33 g serving)	Median of regular protein substitutes [10]
Energy kcal	103	
Protein g	20	20
Carbohydrate g	5.4	
Fibre g	0.25	
Fat g	0.33	
DHA mg	100	

Minerals		
Sodium mg	9.9	202
Potassium mg	200	332
Chloride mg	–	216
Calcium mg	840	470
Phosphorus mg	–	356
Magnesium mg	8.2	109.6

Trace elements		
Iron mg	10	7.2
Zinc mg	10	6.4
Iodine μg	124	84.6
Manganese mg	1.8	1
Copper mg	–	0.8
Molybdenum μg	35	25.8
Selenium μg	25	27.8
Chromium μg	32	20

Vitamins		
Vitamin A μg	730	318
Vitamin E mg	7	5.2
Vitamin D3 μg	14	8
Thiamin mg	0.8	0.6
Riboflavin mg	1.0	0.8
Pyridoxine mg	0.8	0.6
Vitamin B12 μg	2.2	1.6
Vitamin C mg	70	36.6
Vitamin K1 μg	35	26
Folic acid μg	124	103.6
Biotin μg	23	16
Niacin mg	7.6	8
Pantothenic acid mg	3	2.6

Other nutrients		
Inositol mg	90	–
Choline mg	200	200

Essential and conditionally essential amino acids		

## Data Availability

The data presented in this study are available on request from the corresponding author.
